# Analysis of Volatile Molecules Present in the Secretome of the Fungal Pathogen *Candida glabrata*

**DOI:** 10.3390/molecules26133881

**Published:** 2021-06-25

**Authors:** Juan Ernesto López-Ramos, Elihú Bautista, Guadalupe Gutiérrez-Escobedo, Gabriela Mancilla-Montelongo, Irene Castaño, Marco Martín González-Chávez, Alejandro De Las Peñas

**Affiliations:** 1 IPICYT, División de Biología Molecular, Instituto Potosino de Investigación Científica y Tecnológica, Camino a la Presa San José, #2055, Col. Lomas 4ª Sección, San Luis Potosí CP 78216, San Luis Potosí, Mexico; ernesto.lopez@ipicyt.edu.mx (J.E.L.-R.); maria.gutierrez@ipicyt.edu.mx (G.G.-E.); icastano@ipicyt.edu.mx (I.C.); 2 IPICYT, CONACYT-Consorcio de Investigación, Innovación y Desarrollo para las Zonas Áridas, Instituto Potosino de Investigación Científica y Tecnológica A. C, Camino a la Presa San José, #2055, Col. Lomas 4ª Sección, San Luis Potosí CP 78216, San Luis Potosí, Mexico; francisco.bautista@ipicyt.edu.mx; 3 CONACYT, Facultad de Medicina Veterinaria y Zootecnia, Universidad Autónoma de Yucatán, Carretera Mérida-Xmatkuil Km 15.5 S/N, Mérida CP 97100, Yucatán, Mexico; maria.mancilla@correo.uady.mx; 4 Facultad de Ciencias Químicas, Av. Dr Manuel Nava 6, Zona Universitaria, Universidad Autónoma de San Luis Potosí, San Luis Potosí CP 78290, San Luis Potosí, Mexico; gcmm@uaslp.mx

**Keywords:** secretome, *Candida* *glabrata*, secretome, GC-MS analysis, phenylethanol, eicosane, nonanoic acid

## Abstract

*Candida albicans, Candida glabrata*, *Candida parapsilosis* and *Candida tropicalis* are the four most common human fungal pathogens isolated that can cause superficial and invasive infections. It has been shown that specific metabolites present in the secretomes of these fungal pathogens are important for their virulence. *C. glabrata* is the second most common isolate world-wide and has an innate resistance to azoles, xenobiotics and oxidative stress that allows this fungal pathogen to evade the immune response and persist within the host. Here, we analyzed and compared the *C. glabrata* secretome with those of *C. albicans, C. parapsilosis, C. tropicalis* and the non-pathogenic yeast *Saccharomyces cerevisiae*. In *C. glabrata*, we identified a different number of metabolites depending on the growth media: 12 in synthetic complete media (SC), 27 in SC-glutamic acid and 23 in rich media (YPD). *C. glabrata* specific metabolites are 1-dodecene (0.09 ± 0.11%), 2,5-dimethylundecane (1.01 ± 0.19%), 3,7-dimethyldecane (0.14 ± 0.15%), and octadecane (0.4 ± 0.53%). The metabolites that are shared with *C. albicans, C. glabrata*, *C. parapsilosis,* *C. tropicalis* and *S. cerevisiae* are phenylethanol, which is synthesized from phenylalanine, and eicosane and nonanoic acid (identified as trimethylsilyl ester), which are synthesized from fatty acid metabolism. Phenylethanol is the most abundant metabolite in all fungi tested: 26.36 ± 17.42% (*C. glabrata*), 46.77 ± 15.58% (*C. albicans*), 49.76 ± 18.43% (*C. tropicalis*), 5.72 ± 0.66% (*C. parapsilosis*.) and 44.58 ± 27.91% (*S. cerevisiae*). The analysis of *C. glabrata’s* secretome will allow us to further our understanding of the possible role these metabolites could play in its virulence.

## 1. Introduction

*Candida* spp. are the main cause of fungal infections in immunocompromised patients. These fungal pathogens can cause superficial and invasive infections [1]. In the US, these infections are caused by *Candida albicans* (39%), *Candida glabrata* (28%), *Candida parapsilosis* (15%) and *Candida tropicalis* (9%) [2].

It has been shown that diverse microbial pathogens have signaling mechanisms within the cell population for intercellular communication, which helps to develop a successful infection [3]. The secreted molecules constitute the secretome of these microbial pathogens and have a direct effect on cells in the population and other competing microbes. *Saccharomyces cerevisiae*, a non-pathogenic yeast closely related phylogenetically to *C. glabrata*, secretes phenylethanol, tyrosol and tryptophol to the environment and this induces filamentation in other nearby cells [4]. The synthesis of these aromatic alcohols depends on Aro8, Aro9 and Aro10 [5]. In *C. albicans*, it has been shown that the secreted farnesol can induce resistance to oxidative stress [6]. Resistance to the oxidative stress response is an important virulence factor in *C. albicans*.

In the last decade, *C. glabrata* has emerged as the second cause of candidiasis [7] and has been associated with high mortality in immunocompromised patients [8]. *C. glabrata* must be able to detect extracellular signals in the environment such as nutrient availability, presence of xenobiotics or reactive oxygen species, in order to reprogram gene expression to respond and adapt to the new environment within the host. The *C. glabrata* secretome has not been fully described. However, it is conceivable that specific molecules present in the secretome could modulate *C. glabrata* virulence factors such as: (a) adherence to host cells [9], (b) biofilm formation [10,11], (c) resistance to xenobiotics [12,13], and (d) the oxidative stress response, which is mediated by enzymatic (catalase, superoxide dismutases, sulfiredoxin, peroxiredoxins) and non-enzymatic (glutathione) mechanisms [14,15].

Recently, *C. glabrata* secreted proteomes and the secreted metabolite profiles from clinical isolates of *Candida* spp. from different sites (blood, respiratory tract and vulvovaginal) have been reported. These analyses have been carried out by liquid chromatography-tandem mass spectrometry (LC-MS/MS), matrix-assisted laser desorption/ionization time-of-flight mass spectrometry (MALDI-TOFMS) and nuclear magnetic resonance (NMR) [16,17]. Furthermore, the presence of small molecular weight volatile molecules has been described from the *Candida auris* and *C. albicans* secretome by gas chromatography-mass spectrometry (GC-MS). GC-MS has the advantage that it can provide a quantitative analysis of volatile compounds from biological samples [18].

Here, we identified by GC-MS small molecular weight volatile molecules from *C. glabrata, C. albicans, C. parapsilosis, C. tropicalis* and *S. cerevisiae* secretomes. We made a comparative analysis and found that the main molecules shared by these yeasts are derived from fatty acid and aromatic alcohol metabolism. In addition, we identified 1-dodecene, 2,5-dimethylundecane, 3,7-dimethyldecane and octadecane as specific metabolites present in the secretome of *C. glabrata*.

## 2. Results

**Figure 1:**
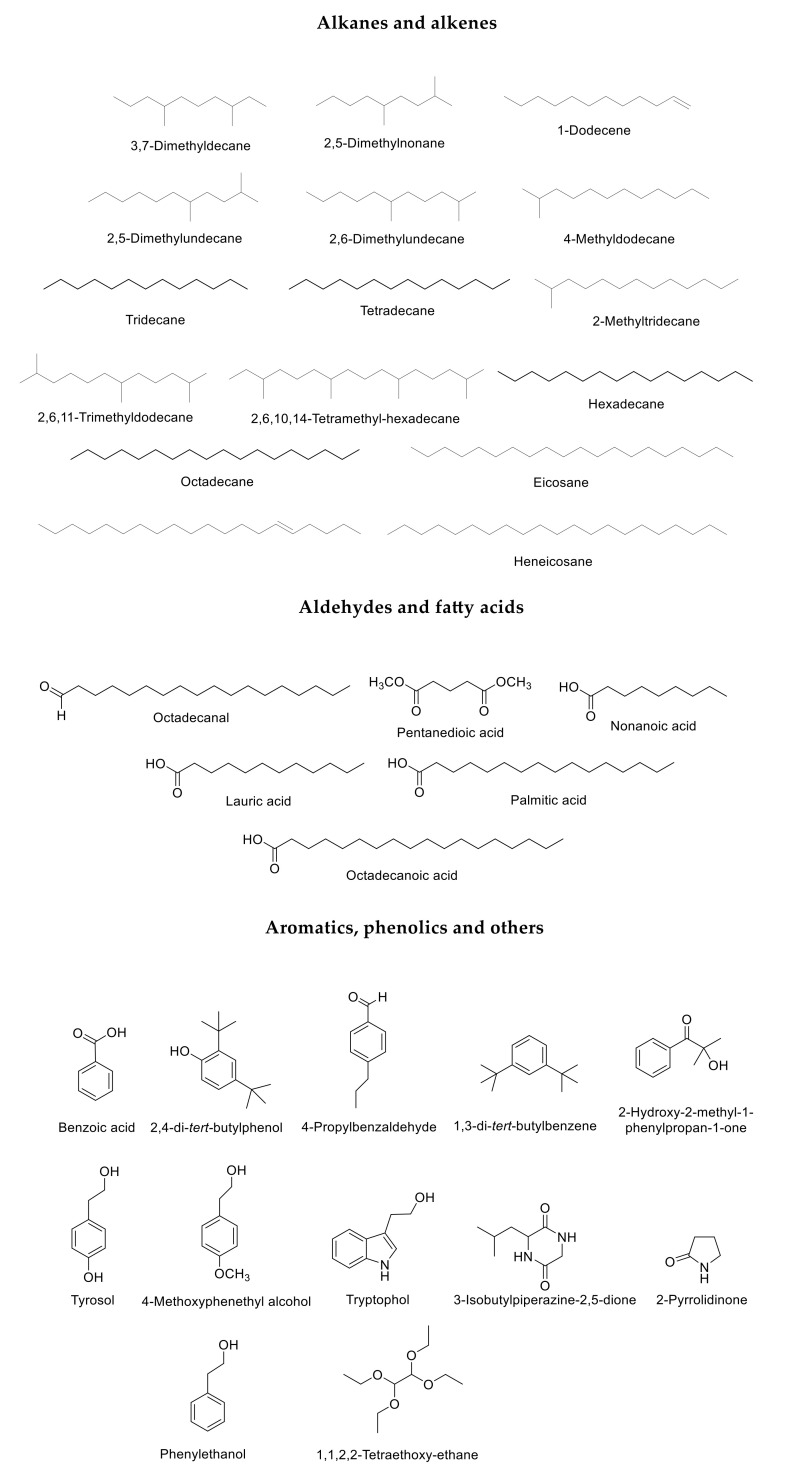
C. glabrata, C. albicans, C. parapsilosis, C. tropicalis and S. cerevisiae metabolites identified by GC-MS.

**Figure 2:**
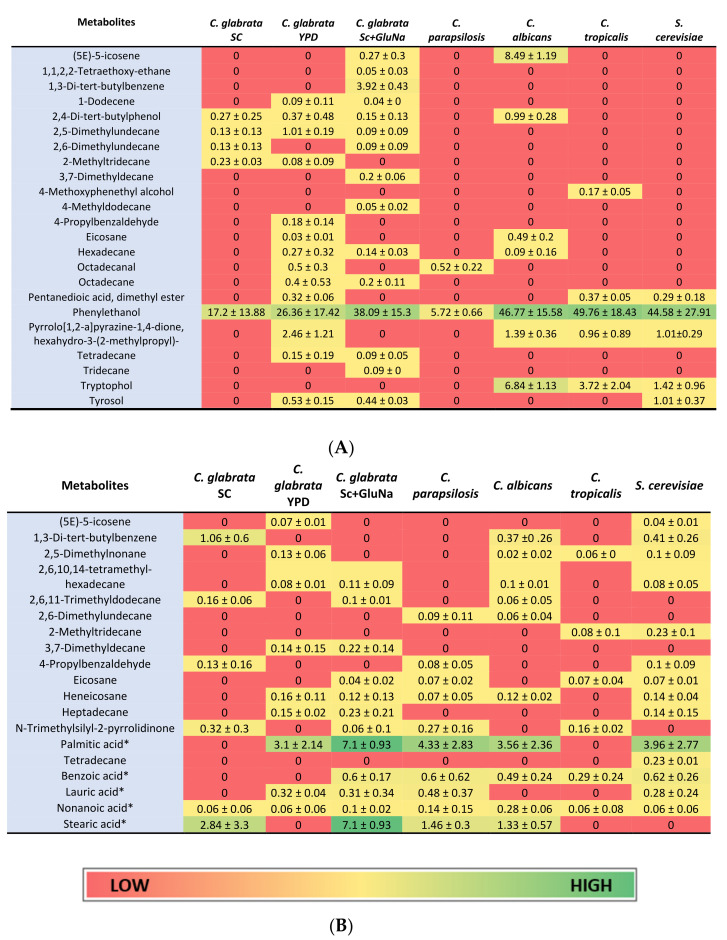
Heatmap of the main metabolites identified in *C. glabrata, C. albicans, C. parapsilosis, C. tropicalis* and *S. cerevisiae*. (**A**) Average of relative percentage of metabolites identified by GC-MS without derivative treatment. (**B**) Average of relative percentage of metabolites identified by the derivatization process prior to GC-MS analysis. Numbers are the average of the relative percentage of a given metabolite of four experimental replicates from three independent biological experiments and are displayed as colors ranging from red (low) to green (high). The relative percentage of a metabolite is represented as a ratio to the total area of all detected metabolites. *C. parapsilosis, C. albicans, C. tropicalis, S. cerevisiae* metabolites were obtained from cells grown on YPD. *C. glabrata* metabolites were obtained from cells grown on YPD, SC and SC+GluNa (see Section 4). * Identified as silylated derivative.

The *C. glabrata* secretome has not been fully described and the identity of theses metabolites could be important for its virulence. To identify the molecules present in the secretome of *C. glabrata, C. albicans, C. tropicalis, C. parapsilosis* and *S. cerevisiae* (all strains are described in Table 1, see Section 4), cells were grown in YPD (rich media, *Cg*, *Ca*, *Ct*, *Cp* and *Sc*), SC (Synthetic complete media, *Cg*) and SC + GluNa (Synthetic complete media with glutamic acid as nitrogen source, *Cg*) and extracts from the cell-free supernatant were obtained from each culture and analyzed by GC-MS (see Section 4). The metabolites were identified and classified in three groups based on their biosynthetic origin: alkanes and alkenes, aldehydes and fatty acids and aromatics and phenolics (Figure 1). To ensure that the molecules identified were specific for each strain, the metabolites present in the media without cells were subtracted from the cell-free supernatant. We identified metabolites present in the *C. glabrata, C. parapsilosis, C. albicans, C. tropicalis,* and *S. cerevisiae* secretome grown in YPD: 23 in *C. glabrata*, 12 in *C. parapsilosis*, 20 in *C. albicans*, 12 in *C. tropicalis*, and 20 in *S. cerevisiae* (Figure 2). In *C. glabrata*, 27 metabolites were identified in SC + GluNa and 12 in SC media (Figure 2). Four metabolites were present only in *C. glabrata*: 1-dodecene (0.09 ± 0.11%), 2,5-dimethylundecane (1.01 ± 0.19%), 3,7-dimethyldecane (0.4 ± 0.53%) and octadecane (0.14 ± 0.15%) (Figure 2) [18].

Metabolites were classified according to their structures into three groups: alkanes and alkenes, aldehydes and fatty acids and aromatics, phenolics and others (see Section 4).

**Figure 3:**
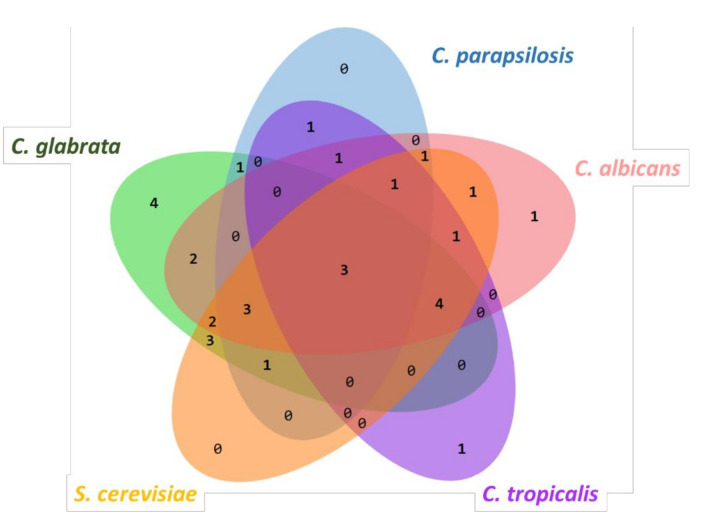
Venn diagram analysis of shared and specific metabolites in *C. glabrata, C. albicans, C. parapsilosis, C. tropicalis* and *S. cerevisiae*.

To determine which metabolites are shared by *C. glabrata, C. parapsilosis, C. albicans, C. tropicalis* and *S. cerevisiae*, we constructed a Venn Diagram (Figure 3) [19]. There are three molecules that are present in all of these fungal microorganisms: phenylethanol (*Cg:* 26.36 ± 17.42%, *Cp:* 5.72 ± 0.66%, *Ca*: 46.77 ± 15.58%, *Ct*: 49.76 ± 18.43%, and Sc: 44.58 ± 27.91%), nonanoic acid (*Cg:* 0.06 ± 0.06%, *Cp:* 0.14 ± 0.15%, *Ca:* 0.28 ± 0.06%, *Ct:* 0.06 ± 0.08%, *Sc:* 0.06 ± 0.06%), and eicosane (*Cg:* 0.03 ± 0.01%, *Cp:* 0.07 ± 0.02%, *Ca:* 0.49 ± 0.2%, *Ct:* 0.07 ± 0.04%, *Sc:* 0.07 ± 0.01%). The most abundant molecule shared by these microorganisms is phenylethanol. Both nonanoic acid and eicosane are products of fatty acid metabolism (Figure 3).

**Figure 4:**
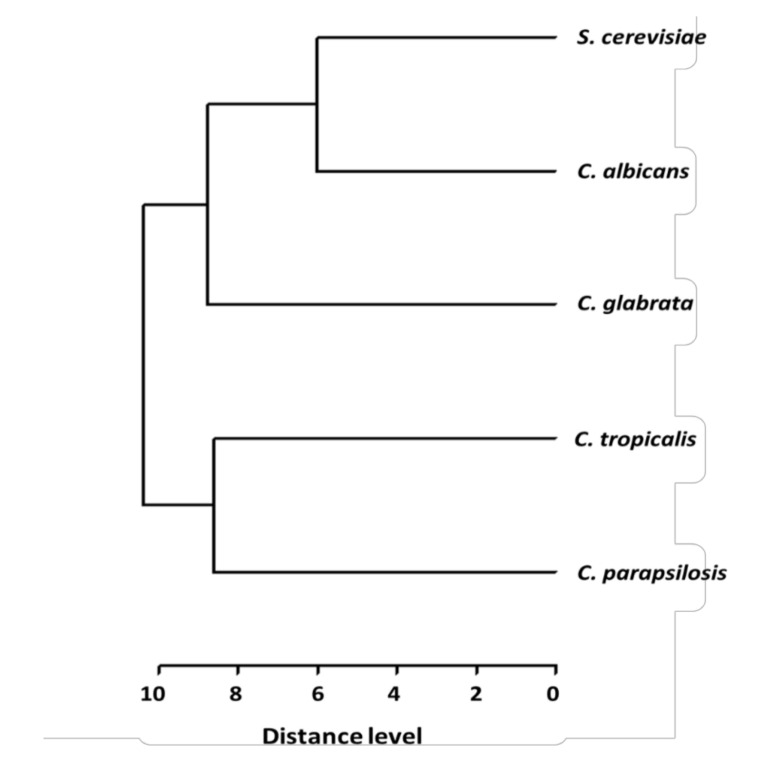
Cluster analysis (CA) of the main metabolites from *C. glabrata, C. albicans, C. parapsilosis, C. tropicalis* and *S. cerevisiae.* The dendrogram depicts the relative distance between *Candida* strains and *S. cerevisiae*. Zero indicates complete similarity and 10 indicates minimal similarity (see Section 4).

**Figure 5:**
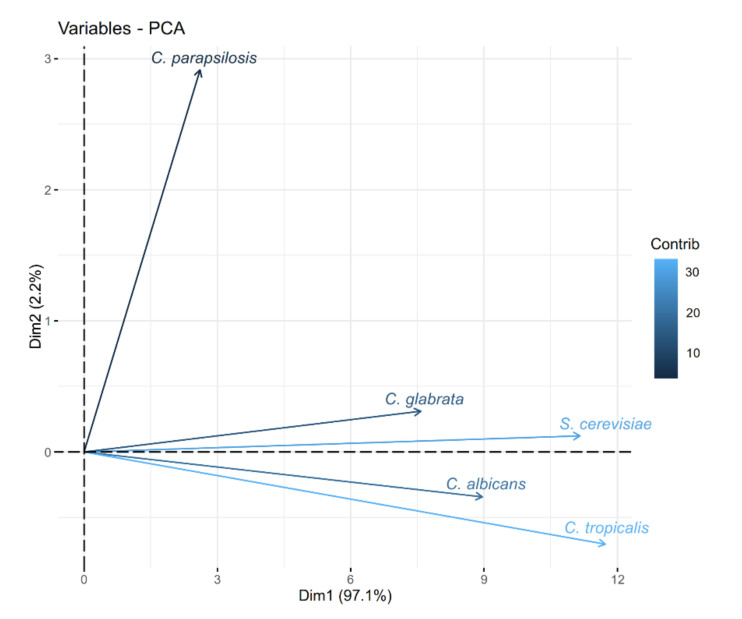
Bi-plot of data from Principal Component Analysis (PCA) for metabolites identified in *C. glabrata, C. albicans, C. parapsilosis, C. tropicalis* and *S. cerevisiae* (see Section 4).

*C. glabrata* and *S. cerevisiae* are closely related phylogenetically and are classified in the *Saccharomycetina* clade. In contrast, *C. albicans*, *C. parapsilosis* and *C. tropicalis* are grouped in the CTG clade and are distantly related to the *Saccharomycetina* clade. To further understand the relation between the metabolites identified in each fungal microorganism, we constructed a cluster analysis (CA) and a principal component analysis (PCA) based on the presence or absence of the metabolites identified for each secretome [17,20,21]. The CA analysis shows that the metabolites are clustered in two groups: (a) metabolites present in *S. cerevisiae*, *C. albicans* and *C. glabrata*, and (b) metabolites present in *C. parapsilosis* and *C. tropicalis* (Figure 4). *C. glabrata* and *S. cerevisiae* share the highest number of metabolites (16 metabolites). Interestingly, 16 metabolites are also shared between *S. cerevisiae* and *C. albicans*, where heptadecane, tetradecane lauric acid (identified as trimethylsilyl laurate) and tyrosol are unique in this group. These metabolites are different from those shared between *S. cerevisiae* and *C. glabrata*. Metabolites only shared between *S. cerevisiae* and *C. glabrata* are 1,3-di-*tert*-butylbenzene, benzoic acid (identified as benzoic acid trimethylsilyl ester), stearic acid (identified as silylated derivative), and tyrosol. Interestingly, *C. glabrata* and *C. albicans* share 15 metabolites. *C. glabrata* unique metabolites are 1-dodecene, 2,5-dimethylundecane, 3,7-dimethyldecane, octadecanal, and octadecane, and for *C. albicans* are 2,6,11-trimethyldodecane and 2,6-dimethylundecane (Figure 2). The PCA also evaluates the relationship between the metabolites identified in *Candida* spp. and *S. cerevisiae* secretomes. This analysis confirms that the metabolites identified in *C. parapsilosis* and *C. tropicalis* are different from those identified in *C. albicans*, *C. glabrata* and *S. cerevisiae* (Figure 5).

Identified metabolites were analyzed by PCA. The contribution of a variable to a given principal component is in percentage. PCA was done using the prcomp function in R and the bi-plot generated using the factoextra package (see Section 4).

**Figure 6:**
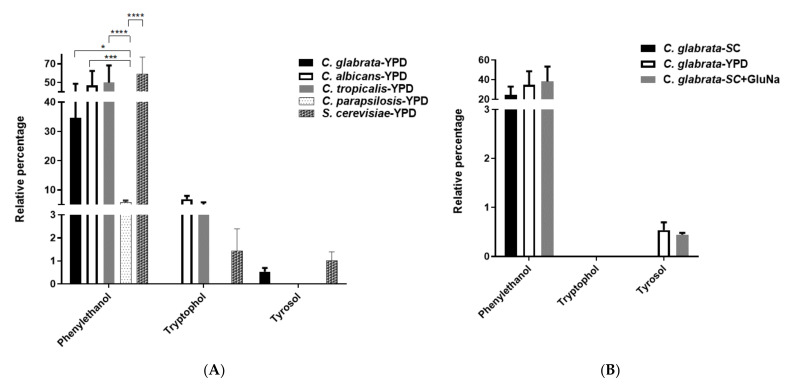
Aromatic alcohols present in the secretome of *C. glabrata, C. albicans, C. parapsilosis, C. tropicalis* and *S. cerevisiae*. Samples were analyzed by GC-MS and relative percentage of phenylethanol, tryptophol and tyrosol are shown. (**A**) Relative percentage of aromatic alcohols in *C. glabrata* grown in YPD, SC and SC + GluNa. (**B**) Relative percentage of aromatic alcohols identified in *C. albicans, C. parapsilosis, C. tropicalis,* and *S. cerevisiae* grown in YPD. If a bar is not present, this indicates the absence of the aromatic alcohol. Data was analyzed using a two-way ANOVA analysis and Tukey’s pos-hoc test. * Indicates *p* < 0.05, *** Indicates *p* < 0.001, **** *p* < 0.0001 (see Section 4).

Phenylethanol, tyrosol and tryptophol have been shown to induce morphological changes in *S. cerevisiae*. We looked for the presence of these aromatic alcohols in the *C. glabrata* secretome in YPD, SC, SC + GluNa media. Phenylethanol is highly abundant in all three media used and tyrosol is not present in SC media. Surprisingly, we did not find tryptophol in the *C. glabrata* secretome in any of the media used (Figure 6A). In *C. albicans, C. parapsilosis, C. tropicalis* and *S. cerevisiae* secretomes, phenylethanol is present however, tryptophol is only present in *C. albicans, C. tropicalis* and *S. cerevisiae;* and tyrosol is present in *C. glabrata* and *S. cerevisiae* (Figure 6B).

## 3. Discussion

*Candida* spp. infections can be superficial or invasive and can be life threatening [22]. In the past decade, there has been an increase in candidiasis and candidemia caused by *C. albicans*, *C. glabrata, C. parapsilosis, C. tropicalis* and most recently by *C. auris*. *C. glabrata* is the second most common *Candida* isolate in nosocomial infections caused by fungi [23,24,25]. *C. glabrata* has several virulence factors that allow for a successful infection. *C. glabrata* adheres to biotic and abiotic surfaces, is capable of forming biofilms, and is resistant to xenobiotics and oxidative stress. In order to understand the role of the secretome of *C. glabrata* in virulence, we characterized and analyzed the *C. glabrata* secretome by GC-MS and compared it with *C. tropicalis, C. albicans, C. parapsilosis* and *S. cerevisiae* secretomes [26]. The specific metabolites present in *C. glabrata* are: 1-dodecene (0.09 ± 0.11%), 2,5-dimethylundecane (1.01 ± 0.19%), 3,7-dimethyldecane (0.14 ± 0.15%) and octadecane (0.4 ± 0.53%) and phenylethanol, nonanoic acid and eicosane are shared in *C. glabrata*, *C. tropicalis, C. parapsilosis, C. tropicalis, C. albicans* and *S. cerevisiae* secretomes.

In *S. cerevisiae*, it has been shown that the aromatic alcohols phenylethanol (Phe), tyrosol (Tyr) and tryptophol (Tryp) present in their secretomes can induce filamentation [27,28]. The synthesis of Phe, Tyr and Tryp requires *Sc*Aro8 (aromatic aminotransferase (i)), *Sc*Aro9 (aromatic aminotransferase (ii)) and *Sc*Aro10 (phenylpyruvate decarboxylase), and in addition, these enzymes are involved in the biosynthesis of aromatic amino acids [4,29,30,31]. The expression of *ScARO8*, *ScARO9* and *ScARO10* is repressed by (NH_4_)_2_SO_4_, thus abrogating the biosynthesis of theses aromatic alcohols [27]. In *C. glabrata*, *Cg*Aro8, *CgAro9* and *Cg*Aro10 may play a similar role in the synthesis of Phe and Tyr. The concentration of these aromatic alcohols is reduced in SC + (NH_4_)_2_SO_4_ compared to SC + GluNa media (Figure 2). Interestingly, both the concentrations of these aromatic alcohols and additional metabolites are reduced (Figure 2). It is possible that nitrogen catabolite repression controls the expression of several genes encoding enzymes for the biosynthesis of these metabolites [32]. We were expecting to find Tryp in the *C. glabrata* secretome; however, the absence of Tryp could be due to either the methodology we applied to extract the molecules from the secretomes or strain specific differences. 

Nonanoic acid is a 9-carbon fatty acid that has been shown to be present in *Trichoderma harzianum*, an entomopathogenic fungus. This fatty acid inhibits spore formation in *Moniliophthora roreri* and in *Crinipellis perniciosa*, both plant pathogenic fungi and can also inhibit their mycelial growth, but only at high concentrations [33]. In addition, it has been shown that nonanoic acid has antifungal activity against the fungal pathogen *Trichophyton mentagrophytes* and has also been identified in *C. glabrata* [19,34]. 

Eicosane is a 20-carbon alkane. It is a ubiquitous compound present in bacteria, plants and animals. Eicosane has been identified as a component of leaf cuticle in *Agave attenuate* and *Triticum aestivu* [35]. It has also been shown to be present in *Streptomyces cacaoi* and in specific organs of mammals like heart, kidney and in bovine adipocytes [36,37,38]. In human fungal pathogens up to now, eicosane has not been described in *Candida species*. Here, we now show that eicosane is present in *C. glabrata*, *C. tropicalis, C. parapsilosis, C. tropicalis, C. albicans,* and *S. cerevisiae*.

The identification of these three molecules present in *C. glabrata*, *C. tropicalis, C. parapsilosis, C. tropicalis* and *C. albicans* and *Saccharomyces cerevisiae* secretomes will allow us to ask whether these metabolites can modulate virulence in these fungal pathogens: are they signaling molecules that regulate stress responses? Can they induce morphological changes, or do they modify global transcriptional expression?

## 4. Materials and Methods

### 4.1. Strains

**Table 1:** Strains used in this study.

Strains			Source of Reference
*C. glabrata*	BG14	*ura3*Δ:Tn*903* Neo ^R^ Ura^-^	[39]
*C. parapsilosis*	ATCC-22019	Clinical Isolate	Lab. collection
*C. tropicalis*	ATCC-750	Clinical Isolate	Lab. collection
*C. albicans* Sc5314	ATCC MYA2876	Clinical Isolate	Lab. collection
*S. cerevisiae* S288c	ATCC 504208	MATα *SUC2 mal mel gal2 CUP1*	Lab. collection

### 4.2. Media and Growth Conditions

Cells from *Candida glabrata, Candida albicans, Candida tropicalis*, *Candida parapsilosis*, and *Saccharomyces cerevisiae* were grown at 30 °C in: rich (YPD) medium containing yeast extract at 10 g/L, peptone at 20 g/L and 2% dextrose. Synthetic complete (SC) medium contained yeast nitrogen base at 1.7 g/L, (NH_4_)_2_SO_4_ at 5 g/L, 0.6% casamino acids, 2% glucose, and when needed, was supplemented with uracil at 25 mg/L. SC-GluNa medium is SC medium but (NH_4_)_2_SO_4_ was replaced for L-glutamic acid monosodium salt as a nitrogen source (1 g/L). Cells were grown for 72 h at 30 °C in 125 mL in Corning culture flasks. Cell-free supernatants were collected by centrifugation at 1300× *g* and filtration using 25 mm filter paper (Whatman) and syringe filters 0.2 µm (Nalgene Thermo Scientific). Fresh medium without cells was used as control to evaluate the relative concentration of metabolites in the media.

### 4.3. Sample Preparation for GC-MS Analysis 

Samples were non-derivatized and derivatized for the GC-MS analysis. For derivatized samples, the preparation prior to GC-MS analysis was performed according to Semreen [18] methodology as follows: 30 mL from cell-free supernatants were extracted three times with 90 mL of chloroform (Fisher Scientific, Santa Clara, CA, USA). The chloroform layer was dehydrated over anhydrous sodium sulphate (Fisher Scientific), filtered, and followed by evaporation using a rotatory evaporator (Buchi, Essen, Germany). The residue collected from each extract was dissolved in 500 μL of chloroform prior to GC-MS injection. In addition, 100 µL of the chloroform extract was derivatized by adding 50 μL of *N*-trimethylsilyl-*N*-methyl trifluoroacetamide and trimethylchlorosilane (MSTFA + 1% TMS) followed by incubation at 50 °C for 30 min prior to GC-MS injection. 

### 4.4. GC-MS Spectrometry Analysis

The GC-MS analysis was performed using a 7890B Gas Chromatograph (G3440B) coupled to a 5977A (G7039A) mass detector (Agilent Technologies, Santa Clara, CA, USA) with a HP-5ms UI column (30 m long × 0.25 mm internal diameter × 0.25 µm film thickness). The stripping gas used was helium (99.9995% purity) with a flow rate of 0.78371 mL/min. The initial temperature of the column was 70 °C maintained for 10 min followed by an increase of 10 °C/min to reach 200 °C, which was held for 10 min. The injection volume and injection temperature were 0.2 µL and 280 °C using splitless mode. The mass spectrometer operated in electron compact mode with an energy of 70 eV. Both the ion source temperature and the interface temperature were 230 °C and 200 °C respectively. The data acquisition mode was Scan starting at 20 to 500 *m/z*. Data collection and analysis were performed using MSD Enhanced ChemStation Software (Agilent Technologies). Mass spectra were identified by comparing the measured fragmentation patterns with those found in the W10N11 library database.

### 4.5. Statistical Analysis

The relative quantity of each metabolite was represented as relative percentage calculated by considering the area under the peak obtained by GC-MS of each metabolite as a ratio with the total area of all other metabolites. Data represent the mean of the relative percentage of three biological repeats ± SD. Heatmap was generated with Excel (Microsoft Office 2019) according to the relative percentage data of each metabolite [18]. Venn diagrams were performed using an online tool (http://jvenn.toulouse.inra.fr/app/example.html accessed on 28 May 2021) [19]. For the cluster analysis (CA), R software was used, applying Euclidian distance [21]. The principal component analysis (PCA) was done using the prcomp function in R and the bi-plot generated using the factoextra package [17,20,21]. Relative percentage of aromatic alcohols was analyzed using a two-way ANOVA analysis and Tukey’s pos-hoc test (*p* < 0.05). GraphPad Prism 8 was used to perform the analysis.

## 5. Conclusions

For a successful infection, fungal pathogens once engulfed by phagocytic cells, need to respond and adapt to the new environment. Fungal pathogens must detect the new hostile environment and reprogram gene expression in order to activate new pathways to maintain cell wall integrity, respond to xenobiotics, nutritional deprivation and oxidative stress. These responses allow survival and persistence within the host. Secreted metabolites by fungal pathogens have been proposed to be important for virulence. Some metabolites can induce morphological changes like filamentation (Phe, Tyro, Tryp) or induced resistance to oxidative stress (farnesol) in neighboring cells. Here, we identified specific metabolites in *C. glabrata*, *C. tropicalis, C. parapsilosis, C. tropicalis, C. albicans* and *S. cerevisiae* secretomes. These metabolites are products of fatty acid biosynthesis (nonanoic acid and eicosane) and aromatic alcohols (Phe, Tyro, Tryp). It is important to evaluate the biosynthesis pathways of theses metabolites and whether they can modulate the expression of stress response pathways (cell wall integrity, nutritional deprivation and resistance to xenobiotics and oxidative stress) as virulence factors in these fungal pathogens.

## Data Availability

Not applicable.
